# Irrigation with Bupivacaine at the Surgical Bed for Postoperative Shoulder Tip and Abdominal Pain Relief after Laparoscopic Cholecystectomy

**DOI:** 10.1055/s-0042-1758141

**Published:** 2022-11-06

**Authors:** Affan Iqbal, Amir Khodavirdipour, HR Ravishankar

**Affiliations:** 1 Department of General Surgery, Sagar Hospitals, Bangalore, Karnataka, India; 2 Department of General Surgery, Seventh-Day Adventist Hospital, Bangalore, Karnataka, India; 3 School of Sport and Health Sciences, University of Brighton, Brighton, United Kingdom.

**Keywords:** cholecystectomy, gallstone, bupivacaine, laparoscopy

## Abstract

Gallstones in western countries are primarily composed of cholesterol. However, mixed or pigment stones, which contain a higher proportion of bilirubin, are more frequently seen in developing nations and Asia than in western countries. Abdominal and shoulder tip pains (STPs) are common complaints following the standard laparoscopic cholecystectomy procedure. To date, all pain management modalities have proven variable outcomes. This prospective randomized study included 82 patients who underwent elective laparoscopic cholecystectomy. The control group received 20 mL of normal saline, whereas the study group received a 20-mL instillation of 0.5% bupivacaine at the gallbladder bed after surgical resection. The Visual Analog Scale (VAS) was used to analyze abdominal pain and STP. The mean age ranged from 20 to 80 years. Abdominal VAS at 6, 12, 18, 24, 30, 36, and 48 hours were statistically insignificant. The majority were discharged on postoperative day 1 (32 studies, 37 control). Follow-up VAS after 1 week for STP VAS and abdominal pain VAS in both groups were statistically insignificant. Even with small numbers of a well-conducted randomized trial, we demonstrated that bupivacaine irrigation at the gallbladder bedpost laparoscopic cholecystectomy does not affect pain relief.

## Introduction

Laparoscopic cholecystectomy (LC) is the gold standard in addressing benign gallbladder disease 1 and is one of the most widely performed surgeries in a general surgery department in India. The most common indications for LC as gallbladder stones are seldomly performed for carcinoma gallbladder. 2 LC is a minimally invasive surgery with reduced morbidity compared with its open counterpart; however, it has a few disturbing postoperative adverse effects, such as shoulder tip pain (STP), abdominal pain, port site pain, infection, granuloma, and hernia.

Visceral pain is caused by intra-abdominal cavity content stretching and peritoneal inflammation. Furthermore, phrenic nerve irritation is caused by residual carbon dioxide. Postoperative abdominal pain develops within the first 24 hours, and STP usually manifests only on the second day. 2 Analgesics and opioids used in pain management have been reported to show variable results. 1 Pain is derived from various situations, namely, surgical site pain (somatic), deep visceral pain, and STP due to phrenic nerve irritation. Evidence suggests that in 17 to 41% of patients, the most probable cause of prolonged hospital stay is due to pain. 1 Postoperative pain is complex after laparoscopic surgery; hence, various specialties suggest an effective treatment to alleviate pain through multimodal support, 1 which includes desensitizing the sensory afferents (adequately injecting the skin with a local anesthetic [LA] before proceeding with an incision). Opioid administration preoperatively, a LA flushed into the peritoneal cavity, intravenous fluids, and electrolytes were adequately given. 1 Few studies showed that bupivacaine irrigation over the surgical bed proved effective in reducing pain in the first few hours after LC. 1 2 3 Whereas another study did not have such an effect. 4

Abdominal pain and dyspepsia were the most common presenting symptoms occurring, either alone or together, in patients with gallbladder stones. Many other studies revealed these two symptoms as the most commonly reported symptoms among patients with cholelithiasis. 5 Ursodeoxycholic acid, used for cholesterol stone diseases, has not been proven successful and is not usually applicable to all patients. 6 Additionally, gallbladder elevations, called polyps, that protrude onto the lumen most of the time were observed with a higher gallbladder cancer rate in the Indian ethnicity shown in a study including 2,359 patients with gallbladder polyps (multivariate analysis: 5.5% vs. 0.08%). 7 8 A study shows that intraperitoneal irrigation of the diaphragmatic surface and gallbladder fossa with bupivacaine/lignocaine and normal saline may effectively control visceral abdominal pain after LC. 9 Nonsteroidal anti-inflammatory drugs, wound infiltration with LA, and intermittent intramuscular narcotics are various methods utilized for pain management with variable success after laparoscopic surgeries. 10

## Materials and Methods

**Fig. 1:**
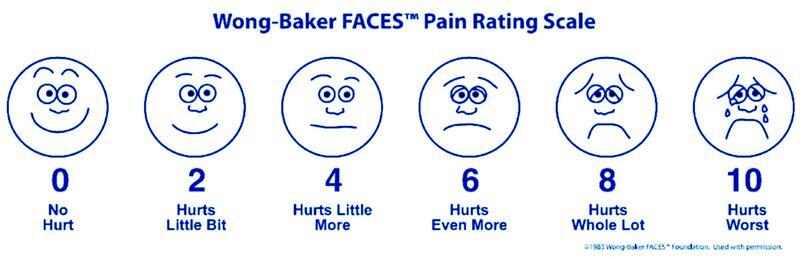
Wong–Baker pain rating scale.

This prospective randomized study was conducted in the Department of General Surgery, Sagar Hospitals, Bangalore, Karnataka, India over 18 months, which included 82 patients who were admitted and subsequently underwent elective standard four-port LC. The patients had their gallstones imaged by ultrasound before surgery ward admission and were divided randomly by a computer-generated table into two groups (42 studies and 40 control, Table 1 ). The control group received 20 mL of normal saline and the study group received a 20-mL instillation of 0.5% bupivacaine at the gallbladder bed after surgical resection. All the patients received pain relief as per the World Health Organization (WHO) pain ladder. Surgery was performed by a senior consultant surgeon with a resident as an assistant. Instillation was done via the lateral most port. Patients were postoperatively analyzed every 6 hours for STP and abdominal pain and were followed up until discharge. Pain assessment was done using the Wong–Bakers Visual Analog Scale (VAS) ( Fig. 1 ). Inclusion criteria are as follows: patients 18 years and older, both symptomatic and asymptomatic patients with cholelithiasis, patients with cholecystitis, a virgin abdomen, and single procedure LC (not with double procedures in the same sitting). Exclusion criteria are as follows: patients with open cholecystectomy, LC converted to open, perforated gallbladder (peritonitis), pregnancy, other abdominal procedures in the same sitting with LC, and abdominal drains. Adequate permission was acquired from the medical ethics committee of the hospital, and all research was done with the informed consent of each patient.

**Table 1:** Sex group cross-tabulation

			Group		Total	*p* -Value
			Control	Study		
Sex	Female	Count	22	22	44	0.82
		% within group	55.0	52.4	53.7	
	Male	Count	18	20	38	
		% within group	45.0	47.6	46.3	
Total		Count	40	42	82	
		% within group	100.0	100.0	100.0	

Note: Chi-square test showing a nonsignificant *p* -value when correlated.

## Results

The collected data was entered in Microsoft Excel and analyzed using the Statistical Package for the Social Sciences version 17. A total of 82 patients were analyzed and formulated. The demographic data were analyzed as frequencies, central tendency measures, and dispersion. The mean age (years) of groups A and B ranged from 20 to 80 years. With the highest age in the fifties (26%) followed by forties (20.7%). STP was experienced by 15 patients at 6 hours, wherein 10 patients had a VAS of 2 (study group = 8, control group = 2), 2 had a VAS of 4 from the control group, and 2 had a VAS of 6 from the study group, without a significant difference in both groups ( *p* = 0.128 and 0.125, respectively). STP was experienced by 13 patients at 12 hours, wherein 9 had a VAS of 2 (study group = 7, control group = 2), 3 had a VAS of 4 (study group = 1, control group 2), and 1 had a VAS of 6 from the control group, without a significant difference in both groups ( *p* = 0.966 and 0.966, respectively). At 18 hours, 8 patients had STP, wherein 5 had a VAS of 2 (4 study, 1 control) and 3 had a VAS of 4 (1 study, 2 control), without significant difference in both groups ( *p* = 0.689 and 0.688, respectively). At 24 hours, 4 patients had STP, wherein 2 had a VAS of 2 from the study group, without a statistically significant difference in both groups ( *p* = 0.400 and 0.435, respectively). The majority were discharged on postoperative day 1 (32 studies, 37 control).

The follow-up VAS after 1 week for STP and abdominal pain were statistically insignificant in both groups.

A total of 82 patients, who planned for elective cholecystectomy, were included in this study and were divided into two groups. All are adult males and females. The gender distribution among the two groups showed a nonsignificant difference (chi-square test *p* = 0.82) ( Table 1 ). The Fisher's exact test was used to study the association between gender and study group, and the Student's *t* -test was used to study the association between study group and age, STP (VAS score), and abdominal pain (VAS score). STP VAS at 30, 36, and 48 hours were statistically insignificant. Abdominal pain VAS at 6, 12, 18, 24, 30, 36, and 48 hours was statistically insignificant ( Table 2 ).

**Table 2:** STP VAS and abdominal pain VAS were statistically insignificant

Variable	*p* -Value
Shoulder tip pain 6 h	0.128
	0.125
@12	0.966
	0.966
@18	0.689
	0.688
@24	0.400
	0.435
@30	0.718
	0.709
@36	0.803
	0.795
@48	0.781
	0.772
Abdomen pain vas 6 h	0.162
	0.163
@12	0.685
	0.685
@18	0.589
	0.589
@24	0.327
	0.334
@30	0.271
	0.245
@36	1.000
	1.000
@42	0.776
	0.611
@48	0.754
	0.746
Follow-up	0.777
	0.777

Abbreviations: STP, shoulder tip pain; VAS, Visual Analog Scale.

## Discussion

Nowadays, LC is one of the common procedures in the general surgery and gastroenterology departments. The learning curve for laparoscopy is long; however, surgical trainees are remarkably exposed to this procedure, thereby making them more comfortable with performing LC than other procedures. 11 Particularly, the postoperative care for these patients must be adequate. This study is conducted to understand the benefits of intraperitoneal bupivacaine irrigation in reducing postoperative abdominal pain and STP. Few study outcomes showed variable results although many studies were of high quality. The present study aimed to obtain high quality evidence for the same. This study showed no particular advantage in the use of bupivacaine.

Radiologic studies demonstrate the presence of pneumoperitoneum for as long as 24 hours after LC, The amount of free gas remaining in the abdominal cavity is usually small and has not been regarded as a cause of postoperative morbidity. Components of post-LC pain include visceral pain (78.33%), parietal pain (70%), and STP (23.33%). Intraperitoneal irrigation of the diaphragmatic surface and gallbladder fossa using normal saline, bupivacaine, or lignocaine may effectively control visceral abdominal pain after LC. 12 A study observed a reduced postoperative STP in minor gynecological surgery after intraperitoneal instillation of LA. 13 With the possibility that a similar beneficial effect might be achieved in LC, several studies were conducted with variable results. 12 This study had a small sample size but was conducted in an organized manner. Only the assistant surgeon was aware of the dosage and drug used for irrigating the gallbladder bed. Anesthesia was given following the standard protocol for each case. Postoperative pain management was in union with intravenous analgesia according to the WHO pain ladder. Each data was recorded by the assistant surgeon of the case. A meta-analysis of all studies of intraperitoneal bupivacaine irrigation is required for a better conclusion on the matter. So far, no adverse effects of bupivacaine irrigation have been recorded; hence, it can be used safely by people without allergies.

In conclusion, the present study had a small sample size; thus, drawing a definitive conclusion was difficult. However, this randomized trial was well-conducted and demonstrated that bupivacaine irrigation at the gallbladder bedpost LC does not affect pain relief. The background that most patients who underwent LC have minimal pain should also be considered; hence, several patients are required to conclude with a suitable outcome to demonstrate a tangible and significant difference between both groups.

**Abbreviations**
LC: Laparoscopic Cholecystectomy
OC: Open Cholecystectomy
LA: Local Anesthesia
STP: Shoulder Tip Pain
VAS: Visual Analog Scale
